# Cytological and genome size data analyzed in a phylogenetic frame: Evolutionary implications concerning *Sisyrinchium* taxa (Iridaceae: Iridoideae)

**DOI:** 10.1590/1678-4685-GMB-2017-0077

**Published:** 2018-03-01

**Authors:** Paula Burchardt, Tatiana T. Souza-Chies, Olivier Chauveau, Sidia M. Callegari-Jacques, Lauís Brisolara-Corrêa, Camila Dellanhese Inácio, Lilian Eggers, Sonja Siljak-Yakovlev, José Marcello Salabert de Campos, Eliane Kaltchuk-Santos

**Affiliations:** 1 Programa de Pós-Graduação em Genética e Biologia Molecular, Universidade Federal do Rio Grande do Sul, Porto Alegre, RS, Brazil; 2 Departamento de Botânica, Instituto de Biociências, Universidade Federal do Rio Grande do Sul, Porto Alegre, RS, Brazil; 3 Programa de Pós-Graduação em Botânica, Instituto de Biociências, Universidade Federal do Rio Grande do Sul, Porto Alegre, RS, Brazil; 4 Departamento de Estatística, Instituto de Matemática e Estatística, Universidade Federal do Rio Grande do Sul, Porto Alegre, RS, Brazil; 5 Ecologie Systématique Evolution, Université Paris-Sud, CNRS, AgroParisTech, Orsay, France; 6 Departamento de Biologia, Instituto de Ciências Biológicas, Universidade Federal de Juiz de Fora, Juiz de Fora, MG, Brazil; 7 Departamento de Genética, Instituto de Biociências, Universidade Federal do Rio Grande do Sul, Porto Alegre, RS, Brazil

**Keywords:** Ancestral chromosome number, DNA content, meiotic behavior, pollen viability, polyploidy

## Abstract

*Sisyrinchium* is the largest genus of Iridaceae in the Americas and has the greatest amount of cytological data available. This study aimed at investigating how genomes evolved in this genus. Chromosome number, genome size and altitude from species of sect. *Viperella* were analyzed in a phylogenetic context. Meiotic and pollen analyses were performed to assess reproductive success of natural populations, especially from those polyploid taxa. Character optimizations revealed that the common ancestor of sect. *Viperella* was probably diploid (2*n* = 2*x* =18) with two subsequent polyplodization events. Total DNA content (2C) varied considerably across the phylogeny with larger genomes detected mainly in polyploid species. Altitude also varied across the phylogeny, however no significant relationship was found between DNA content changes and altitude in our data set. All taxa presented regular meiosis and pollen viability (> 87%), except for *S.* sp. nov. aff. *alatum* (22.70%), suggesting a recent hybrid origin. Chromosome number is mostly constant within this section and polyploidy is the only source of modification. Although 2C varied considerably among the 20 taxa investigated, the diversity observed cannot be attributed only to polyploidy events because large variations of DNA content were also observed among diploids.

## Introduction

Iridaceae is one of the largest Asparagales families (APG IV, 2016), and chromosome number was determined for about 50% of the 2030 species included, although mostly for taxa from the Northern Hemisphere and Africa (Goldblatt and Takei, 1997). Knowledge about cytological characters has been especially helpful for genera circumscription and to understand species relationship in various genera (Goldblatt and Takei, 1997). In this family, there is great karyological variation, comprising karyotype features, basic chromosome number (*x*) and ploidy level. Chromosome heteromorphism and asymmetry are frequently found in several species (Alves *et al.*, 2011). Such karyotypic variations are especially related to polyploidy and descending disploidy, both considered important events for the diversification of subfamily Iridoideae (Goldblatt and Takei, 1997; Moraes *et al.*, 2015).

*Sisyrinchium* L. is the largest genus of Iridaceae in the Americas (Goldblatt and Manning, 2008) and presents a large amount of cytological data, mainly for North American species (Kenton and Heywood, 1984; Kenton *et al.*, 1986; Goldblatt and Takei, 1997). According to the literature, the ancestral basic chromosome number for *Sisyrinchium* is probably *x* = 9 (Goldblatt and Manning, 2008; Chauveau *et al.*, 2011), although other base numbers (*x*_*2*_ = 5, 6, 8, 17) also occur within the genus (Goldblatt and Manning, 2008). All *Sisyrinchium* species from Brazil studied by Souza-Chies *et al.* (2012) had a base chromosome number of *x* = 9, except *Sisyrinchium micranthum* Cav. (*x* = 8; Tacuatiá *et al.*, 2012). Typically, *Sisyrinchium* species have small chromosomes, especially those with *x* = 8 (< 1 mm), hindering karyotype establishment (Kenton *et al.*, 1986).

Similarly to other families of Asparagales, such as Amaryllidaceae and Orchidaceae, Iridaceae present considerable variation in genome size (Bennett and Leitch, 2012) even within genera such as *Sisyrinchium*, the genus with the largest amount of C-value estimates (Goldblatt *et al.*, 1984; Kenton *et al.*, 1986; Moraes *et al.*, 2015). DNA measurements in *Sisyrinchium* have shown that DNA contents are generally low, with haploid genomes (C-values) varying between 0.25 and 4.20 pg (Kenton *et al.*, 1986; Moraes *et al.*, 2015).

Phylogenetic trees have been used, traditionally, to identify patterns of chromosome diversification (Moraes *et al.*, 2012; Koehler *et al.*, 2008). More recently, however, statistical analyses, such as ancestral state reconstruction based on maximum likelihood, have enabled studies of karyotype evolution under a phylogenetic perspective (Escudero *et al.*, 2014; Moraes *et al.*, 2015). Moreover, cytological data have been successfully used to help taxonomic decisions within Iridaceae (De Tullio *et al.*, 2008).

*Sisyrinchium* is taxonomically complex, and a study published recently showed that most of the infrageneric subdivisions recognized for the genus are not monophyletic (Chauveau *et al.*, 2011). This study focused on clade IV of Chauveau *et al.* (2011), which includes species from South America that belong to two different sections *sensu* Ravenna: *Viperella* and *Hydastylus* in part (Ravenna, 2000, 2002). Thus, in an attempt to provide useful information for future taxonomic studies, cytological data were analyzed in a phylogenetic framework.

Considering the scarce cytological information available for *Sisyrinchium* species from Southern Brazil and its relevance to understand Iridaceae evolution, new data were obtained in the present study concerning: (1) chromosome number and ploidy level, (2) genome size (GS), (3) meiotic behaviour and meiotic index, as well as (4) pollen viability and morphology. Chromosome numbers and GS were then analyzed in an evolutionary context and the relationship between DNA content and altitude variations was also tested.

## Material and Methods

### Taxonomic sampling

A total of 43 accessions representing 25 *Sisyrinchium* taxa from clade IV according to Chauveau *et al.* (2011), hereafter named *Viperella*-*Hydastylus* clade, were collected in Brazil from 2006 to 2014. Most species currently recognized in this monophyletic group (see Chauveau *et al.*, 2011) were included in our sampling. All vouchers were deposited in the ICN Herbarium, Instituto de Biociências, Universidade Federal do Rio Grande do Sul, Porto Alegre, RS, Brazil.

Based on Chauveau *et al.* (2011), two members of the sister sect. *Spathirhachis* were selected as outgroup. Plant material was obtained from the wild, except one outgroup species (Table 1). Taxa sampled, accession numbers, collection data, voucher information and identifiers of GenBank sequence records are given in Table 1. Twenty-seven DNA samples representing each taxon included in the study were used to infer phylogenetic relationships, whereas ploidy levels and genome size information were obtained for 19 and 20 taxa, respectively (Table 2). Additionally, meiotic analyses were conducted on seven species of the ingroup (Table S1), while pollen viability and morphology were reported for 12 species (Table 3).

**Table 1:** Voucher information, geographical origin, altitude and GenBank accession numbers of *Sisyrinchium* species sampled.

Species	Sample ID	Geographical origin	Altitude (m)	Voucher	*rpoC1*	*rpoB*	*matK-trnK*	*psbA-trnH*	*trnQ-rps16*	*nad1 2/3*	*nad4 1/2*	ITS
*Outgroup species*												
*Sisyrinchium macrocarpum* Hieron.	SP235	Argentina (cultivated at UPSBG)	1800-3160^(1)^	*Chauveau H09031* (ICN)	HQ606576	HQ606686	HQ606796**	HQ606906	HQ607016	HQ607234	HQ607344	HQ607124
*Sisyrinchium striatum* Sm.	SP844	Chile: Región Metropolitana, Caleu (cultivated at UPSBG)	1000	*Chauveau H11008* (ICN)	KX432394	KX432497	JQ670496*	KF577366	KX432699	KX432802	KX432905	KF577205
*Ingroup species*												
*Sisyrinchium alatum* Hook.	ESC318	Brazil: Santa Catarina, Campo Alegre (26°10’15.2’’S - 49°14’03.6’’W)	975	*Eggers & Souza-Chies 318* (ICN)	MF506968	MF506971	KF577263**	KF577349	MF506976	MF506979	MF506982	KF577188
*Sisyrinchium* sp. nov. aff. *alatum* Hook.	ESC239	Brazil: Paraná, Bituruna (26°05’10.2’’S - 51°39’42.9’’W)	1120^(2)^	*Eggers & Souza-Chies* 239 (ICN)	KX432309	KX432412	KF577236**	KF577322	KX432614	KX432717	KX432820	KF577161
*Sisyrinchium* sp. nov. aff. *alatum* Hook.	ESC232	Brazil: Santa Catarina, Irani (27°00’21.4’’S - 51°52’25.2’’W)	1088^(3)^	*Eggers & Souza-Chies* 232 (ICN)	NA	NA	NA	NA	NA	NA	NA	NA
*Sisyrinchium balansae* Baker	ESC464	Brazil: Rio Grande do Sul, São Francisco de Paula (29°29’11.9’’S - 50°13’15.6’’W)	895	*Eggers & Souza-Chies 464* (ICN)	KX432312	KX432415	KX432506**	KX432560	KX432617	KX432720	KX432823	KX432919
*Sisyrinchium brasiliense* (Ravenna) Ravenna	ESC379	Brazil: Paraná, Guarapuava (25°24’13.3’’S - 51°43’44.5’’W)	978	*Eggers & Souza-Chies 379* (ICN)	HQ606550	HQ606660	HQ606770**	HQ606880	HQ606990	HQ607208	HQ607318	HQ607099
*Sisyrinchium bromelioides* R.C.Foster ssp. *bromelioides*	ICS140	Brazil: Rio Grande do Sul, Arroio dos Ratos (30°13’18.8’’S - 51°43’21.7’’W)	152	*Inácio et al.*	140 (ICN)	KX432315	KX432418	KX432533*	KX432561	KX432620	KX432723	KX432826
*Sisyrinchium caeteanum* Ravenna	ESC224	Brazil: Santa Catarina, Bom Jardim da Serra (28°23’14.9’’S - 49°33’43.8’’W)	1438	*Eggers & Souza-Chies 224* (ICN)	HQ606537	HQ606647	HQ606757**	HQ606867	HQ606977	HQ607195	HQ607305	HQ607086
*Sisyrinchium coalitum* Ravenna	ESC597	Brazil: Santa Catarina, Ponte Alta do Norte (27°16’26.0’’S - 50°26’26.7’’W)	1052	*Eggers & Souza-Chies 597* (ICN)	KX432320	KX432423	KF577280**	KF577368	KX432625	KX432728	KX432831	KF577207
*Sisyrinchium congestum* Klatt	ICEP230	Brazil: Santa Catarina, Urubici (28°07’14.2’’S - 49°29’15.2’’W)	1738	*Inácio et al.*	230 (ICN)	KX432324	KX432427	KX432534*	KX432564	KX432629	KX432732	KX432835
*Sisyrinchium decumbens* Ravenna	ESC213	Brazil: Rio Grande do Sul, São Francisco de Paula (29°26’44.9’’S - 50°36’17.6’’W)	892^(2)^	*Eggers & Souza-Chies 213* (ICN)	KX432327	KX432430	KX432510**	KX432566	KX432632	KX432735	KX432838	KX432925
*Sisyrinchium decumbens* Ravenna	ESC204	Brazil: Rio Grande do Sul, Cambará do Sul (29°14’26.9’’S - 50°16’07.7’’W)	942	*Eggers & Souza-Chies 204* (ICN)	NA	NA	NA	NA	NA	NA	NA	NA
*Sisyrinchium decumbens* Ravenna	ESC222	Brazil: Rio Grande do Sul, São José dos Ausentes (28°48’06.0’’S - 49°57’10.0’’W)	1133^(2)^	*Eggers & Souza-Chies 222* (ICN)	NA	NA	NA	NA	NA	NA	NA	NA
*Sisyrinchium decumbens* Ravenna	ESC570	Brazil: Rio Grande do Sul, Bom Jesus (28°40’35.3’’S - 50°26’16.3’’W)	1068	*Eggers & Souza-Chies 570* (ICN)	NA	NA	NA	NA	NA	NA	NA	NA
*Sisyrinchium densiflorum* Ravenna	ICEP257	Brazil: Paraná, Ponta Grossa (25°14’38.3’’S - 50°00’41.0’’W)	856	*Inácio et al.*	257 (ICN)	KX432330	KX432433	KX432535*	KX432568	KX432635	KX432738	KX432841
*Sisyrinchium densiflorum* Ravenna	ICEP208	Brazil: Paraná, Campo Alegre (26°10’15.9’’S - 49°14’03.6’’W)	967	*Inácio et al.*	208 (ICN)	NA	NA	NA	NA	NA	NA	NA
*Sisyrinchium flabellatum* Aita & L.Eggers	E693	Brazil: Rio Grande do Sul, Jaquirana (28°55’01.8’’S - 50°18’50.0’’W)	893	*Eggers 693* (ICN)	KX432334	KX432437	KX432513**	KX432572	KX432639	KX432742	KX432845	KX432931
*Sisyrinchium macrocephalum* ssp. *giganteum* Ravenna	ESC382	Brazil: Paraná, Mariopolis (26°22’00.5’’S - 52°31’57.9’’W)	839	*Eggers & Souza-Chies 382* (ICN)	KX432365	KX432468	KF577248**	KF577334	KX432670	KX432773	KX432876	KF577173
*Sisyrinchium macrocephalum* ssp. *giganteum* Ravenna	ESC567	Brazil: Rio Grande do Sul, São Francisco de Paula (29°05’55.7’’S - 52°37’49.7’’W)	872	*Eggers & Souza-Chies 567* (ICN)	NA	NA	NA	NA	NA	NA	NA	NA
*Sisyrinchium* cf. *marchioides* Ravenna	ESC319	Brazil: Santa Catarina, Campo Alegre (26°10’13.9’’S - 49°13’59.6’’W)	971	*Eggers & Souza-Chies 319* (ICN)	MF506969	MF506972	KF577262**	KF577348	MF506977	MF506980	MF506983	KF577187
*Sisyrinchium marginatum* Klatt	ISI143	Brazil: Rio Grande do Sul, Viamão (30°21’50.3’’S - 51°01’44.1’’W)	152	*Inácio et al.*	143 (ICN)	KX432347	KX432450	KX432539*	KX432579	KX432652	KX432755	KX432858
*Sisyrinchium marginatum* Klatt	ESC231	Brazil: Santa Catarina, Curitibanos (27°18’45.4’’S - 50°33’43.1’’W)	1021^(2)^	*Eggers & Souza-Chies 231* (ICN)	NA	NA	NA	NA	NA	NA	NA	NA
*Sisyrinchium marginatum* Klatt	ESC482	Brazil: Rio Grande do Sul, Capão do Leão (31°46’49.1’’S - 52°32’06.1’’W)	76	*Eggers & Souza-Chies 482* (ICN)	NA	NA	NA	NA	NA	NA	NA	NA
*Sisyrinchium marginatum* Klatt	ESC486	Brazil: Rio Grande do Sul, São Lourenço do Sul (31°22’22.2’’S - 52°05’56.1’’W)	53^(2)^	*Eggers & Souza-Chies 486*(ICN)	NA	NA	NA	NA	NA	NA	NA	NA
*Sisyrinchium* sp. nov. aff. *minense* Ravenna	ICEP282	Brazil: Minas Gerais, São Roque de Minas (20°11’28.6’’S - 46°39’37.7’’W)	1377	*Inácio et al.*	282 (ICN)	KX432357	KX432460	KX432540*	KX432581	KX432662	KX432765	KX432868
*Sisyrinchium nidulare* (Hand.-Mazz.) I.M.Johnst.	ESC240	Brazil: Paraná, Pinhão (25°33’52.4’’S - 51°39’30.6’’W)	1000^(2)^	*Eggers & Souza-Chies 240* (ICN)	HQ606522	HQ606632	HQ606742**	HQ606852	HQ606962	HQ607180	HQ607290	HQ607071
*Sisyrinchium nidulare* (Hand.-Mazz.) I.M.Johnst.	ICEP255	Brazil: Paraná, Candoí (25°26’18.2’’S - 51°49’36.6’’W)	977	*Inácio et al.*	255 (ICN)	NA	NA	NA	NA	NA	NA	NA
*Sisyrinchium* sp. nov. aff. *nidulare* (Hand.-Mazz.) I.M.Johnst.	ESC348	Brazil: Paraná, Jaguariaíva (24°21’17.4’’S - 49°48’22.1’’W)	1127	*Eggers & Souza-Chies 348* (ICN)	KX432361	KX432464	KF577245**	KF577331	KX432666	KX432769	KX432872	KF577170
*Sisyrinchium palmifolium* L. ssp. *palmifolium*	ESC255	Brazil: Rio Grande do Sul, Porto Alegre (30°03’33.0’’S - 51°07’03.8’’W)	262^(2)^	*Eggers & Souza-Chies 255* (ICN)	KX432367	KX432470	KF577239**	KF577325	KX432672	KX432775	KX432878	KF577164
*Sisyrinchium palmifolium* L. ssp. *palmifolium*	ESC469	Brazil: Rio Grande do Sul, Aceguá (31°39’00.8’’S - 54°09’09.1’’W)	172	*Eggers & Souza-Chies 469* (ICN)	NA	NA	NA	NA	NA	NA	NA	NA
*Sisyrinchium palmifolium* L. ssp. *palmifolium*	ESC487	Brazil: Rio Grande do Sul, São Gabriel (30°14’40.1’’S - 54°22’13.6’’W)	101	*Eggers & Souza-Chies 487* (ICN)	NA	NA	NA	NA	NA	NA	NA	NA
*Sisyrinchium plicatulum* Ravenna	ESC167	Brazil: Rio Grande do Sul, São Francisco de Paula (29°25’25.7’’S - 50°30’50.4’’W)	866^(2)^	*Eggers & Souza-Chies 167* (ICN)	KX432374	KX432477	KX432523**	KX432588	KX432679	KX432782	KX432885	KX432947
*Sisyrinchium plicatulum* Ravenna	ESC650	Brazil: Santa Catarina, Campo Alegre (26°10’16.5’’S - 49°14’04.3’’W)	1017	*Eggers & Souza-Chies 650* (ICN)	NA	NA	NA	NA	NA	NA	NA	NA
*Sisyrinchium rectilineum* Ravenna	BKF2	Brazil: Rio Grande do Sul, São Francisco de Paula (29°25’26.1’’S - 50°30’50.6’’W)	864	*Burchardtet al.*2 (FUEL)	MF506970	MF506973	MF506974*	MF506975	MF506978	MF506981	MF506984	MF506985
*Sisyrinchium restioides* Spreng.	ESC217	Brazil: Rio Grande do Sul, São Francisco de Paula (29°23’06.0’’S - 50°26’09.0’’W)	889^(2)^	*Eggers & Souza-Chies 217* (ICN)	KX432384	KX432487	KF577235**	KF577321	KX432689	KX432792	KX432895	KF577160
*Sisyrinchium restioides* Spreng.	ESC252	Brazil: Santa Catarina, Correia Pinto (27°37’07.3’’S - 50°20’43.1’’W)	884^(2)^	*Eggers & Souza-Chies 252* (ICN)	NA	NA	NA	NA	NA	NA	NA	NA
*Sisyrinchium vaginatum* Spreng. ssp. *vaginatum*	ESC463	Brazil: Rio Grande do Sul, Pelotas (31°39’16.0"S - 52°28’11.0"W)	85^(2)^	*Eggers & Souza-Chies 463* (ICN)	KX432399	KX432502	KF577277**	KF577364	KX432704	KX432807	KX432910	KF577203
*Sisyrinchium vaginatum* Spreng. ssp. *vaginatum*	ESC471	Brazil: Rio Grande do Sul, Candiota (31°31’20.0"S - 53°30’40.1"W)	394	*Eggers & Souza-Chies 471* (ICN)	NA	NA	NA	NA	NA	NA	NA	NA
*Sisyrinchium vaginatum* Spreng. ssp. *vaginatum*	ESC563	Brazil: Rio Grande do Sul, Caxias do Sul (29°04’00.4"S - 50°58’31.5"W)	835	*Eggers & Souza-Chies 563* (ICN)	NA	NA	NA	NA	NA	NA	NA	NA
*Sisyrinchium weirii* Baker	ESC248	Brazil: Paraná, Palmeira (25°26’05.4"S - 49°50’54.5"W)	948^(2)^	*Eggers & Souza-Chies 248* (ICN)	HQ606535	HQ606645	HQ606755**	HQ606865	HQ606975	HQ607193	HQ607303	HQ607084
*Sisyrinchium weirii* Baker	ESC359	Brazil: Paraná, Balsa Nova (25°27’55.8"S - 49°44’54.1"W)	1048	*Eggers & Souza-Chies 359* (ICN)	NA	NA	NA	NA	NA	NA	NA	NA
*Sisyrinchium wettsteinii* Hand.-Mazz.	ICEP224	Brazil: Santa Catarina, Água Doce (26°44’38.4"S - 51°39’27.7"W)	1284	*Inácio et al.*	224 (ICN)	KX432401	KX432504	KX432553*	KX432603	KX432706	KX432809	KX432912
*Sisyrinchium wettsteinii* Hand.-Mazz.	ESC405	Brazil: Santa Catarina, Santa Cecília (26°46’22.5"S - 50°20’59.8"W)	1158	*Eggers & Souza-Chies 405* (ICN)	NA	NA	NA	NA	NA	NA	NA	NA
*Sisyrinchium* sp. nov. I258	ICEP258	Brazil: Paraná, Ponta Grossa (25°14’38.3’’S - 50°00’41.0’’W)	856	*Inácio et al.*	258 (ICN)	KX432402	KX432505	KX432554*	KX432604	KX432707	KX432810	KX432913

*Notes*: ^(1)^ = altitude from Ravenna (2001) and the following vouchers: Cocucci *et al.*, 2198 (CORD), Cocucci *et al.*, 2246 (CORD), Cocucci *et al.*, 2236 (CORD), Kiesling *et al.*, 6277 (SI), Kiesling *et al.*, 9372 (SI), Haene 1074B (MO); ^(2)^ = altitude from GoogleEarth; * = matK gene (partial coding sequence) and trnK gene (intron partial sequence); ** = matK gene (partial coding sequence); CORD = Herbarium of the IMBIV-Museo Botánico (Argentina); FUEL = Herbarium of the State University of Londrina (Brazil); ICN = Herbarium of the Federal University of Rio Grande do Sul (Brazil); MO = Herbarium of the Missouri Botanical Garden (U.S.A.); SI = Herbarium of the Instituto de Botánica Darwinion (Argentina); UPSBG = Botanical Garden of the University Paris-Sud (France); NA = not available.

**Table 2:** Accession numbers, sampling for phylogenetic analyses, chromosome numbers, ploidy levels, 2C DNA content (pg and Mbp) and monoploid genome sizes (1C*x* value) of *Sisyrinchium* species included in the study.

Species	Sample ID	DNA sequences	2*n* (ploidy level)	2C (pg)	2C (Mbp)	1C*x* (pg)
***Outgroup species***						
*S. macrocarpum*	SP235	X	18 (2*x*)	2.19	2142	1.09
*S. striatum*	SP844	X	18 (2*x*)	2.71	2650	1.35
***Ingroup species***						
*S. alatum*	ESC318	X	36 (4*x*)	7.30	7139	1.83
*S.* subsp. nov. aff*. alatum*	ESC239	X	18 (2*x*)	4.03	3941	2.01
*S.* subsp. nov. aff*. alatum*	ESC232	NA	18 (2*x*)	NA	NA	NA
*S. balansae*	ESC464	X	18 (2*x*)	2.55	2494	1.28
*S. brasiliense*	ESC379	X	NA	4.68	4577	NA
*S. bromelioides* ssp. *bromelioides*	ISC140	X	NA	NA	NA	NA
*S. caeteanum*	ESC224	X	NA	8.17	7990	NA
*S. coalitum*	ESC597	X	NA	NA	NA	NA
*S. congestum*	ICEP230	X	NA	NA	NA	NA
*S. decumbens*	ESC213	X	18 (2*x*)	2.08	2034	1.04
*S. decumbens*	ESC204	NA	18 (2*x*)	2.71	2650	1.35
*S. decumbens*	ESC222	NA	18 (2*x*)	2.65	2592	1.32
*S. decumbens*	ESC570	NA	18 (2*x*)	2.64	2582	1.32
*S. densiflorum*	ICEP257	X	NA	NA	NA	NA
*S. densiflorum*	ICEP208	NA	18 (2*x*)	3.84	3755	1.92
*S. flabellatum*	E693	X	18 (2*x*)	2.99	2924	1.49
*S. macrocephalum* subsp. *giganteum*	ESC382	X	18 (2*x*)	4.80	4694	2.40
*S. macrocephalum* subsp. *giganteum*	ESC567	NA	18 (2*x*)	4.76	4655	2.38
S. cf. *marchioides*	ESC319	X	36 (4*x*)	5.69	5565	1.42
*S. marginatum*	ISI143	X	18 (2*x*)	4.90	4792	2.45
*S. marginatum*	ESC231	NA	18 (2*x*)	NA	NA	NA
*S. marginatum*	ESC482	NA	18 (2*x*)	NA	NA	NA
*S. marginatum*	ESC486	NA	18 (2*x*)	NA	NA	NA
*S.* sp. nov. aff. *minense*	ICEP282	X	NA	NA	NA	NA
*S. nidulare*	ICEP255	NA	18 (2*x*)	3.75	3667	1.87
*S. nidulare*	ESC240	X	18 (2*x*)	3.34	3266	1.67
*S.* sp. nov. aff. *nidulare*	ESC348	X	NA	NA	NA	NA
*S. palmifolium* subsp*. palmifolium*	ESC255	X	18 (2*x*)	4.74	4636	2.37
*S. palmifolium* subsp*. palmifolium*	ESC469	NA	18 (2*x*)	NA	NA	NA
*S. palmifolium* subsp*. palmifolium*	ESC487	NA	18 (2*x*)	4.75	4645	2.38
*S. plicatulum*	ESC167	X	18 (2*x*)	4.85	4743	2.42
*S. plicatulum*	ESC650	NA	18 (2*x*)	NA	NA	NA
*S. rectilineum*	BKF 2	X	18 (2*x*)	5.30	5183	2.65
*S. restioides*	ESC217	X	18 (2*x*)	NA	NA	NA
*S. restioides*	ESC252	NA	18 (2*x*)	NA	NA	NA
*S. vaginatum* subsp. *vaginatum*	ESC463	X	18 (2*x*)	2.68	2621	1.34
*S. vaginatum* subsp. *vaginatum*	ESC471	NA	18 (2*x*)	2.60	2542	1.30
*S. vaginatum* subsp. *vaginatum*	ESC563	NA	18 (2*x*)	2.29	2240	1.14
*S. weirii*	ESC248	X	54 (6*x*)	7.77	7599	1.29
*S. weirii*	ESC359	NA	54 (6*x*)	6.96	6807	1.16
*S. wettsteinii*	ICEP224	X	18 (2*x*)	4.35	4254	2.17
*S. wettsteinii*	ESC405	NA	18 (2*x*)	NA	NA	NA
*S.* sp. nov. I258	ICEP258	X	NA	NA	NA	NA

*Notes*: NA = not available.

**Table 3:** Viability, dimensions and morphology of pollen grains from *Sisyrinchium* species.

Species	Viability		N^*^	Polar axis (P) (μm)	Equatorial diameter (E) (μm)	Ratio (P/E)	Morphology
	N^*^	%^#^		Mean	Mean	Mean	
*S. alatum*	1 (500)	98.50	1 (20)	36.00	43.10	0.84	suboblate
*S. balansae*	1 (500)	99.30	1 (20)	24.80	31.10	0.80	suboblate
*S. decumbens*	9 (4500)	96.33	7 (140)	28.09	31.26	0.90	oblate spheroidal
*S. macrocephalum* subsp. *giganteum*	5 (2500)	99.08	5 (100)	26.12	31.41	0.83	suboblate
*S. marginatum*	20 (10000)	93.01	20 (400)	31.42	35.58	0.88	oblate spheroidal
*S. nidulare*	4 (2000)	87.70	4 (80)	31.90	33.75	0.95	oblate spheroidal
*S. palmifolium* subsp. *palmifolium*	5 (2500)	97.28	5 (100)	28.58	32.32	0.89	oblate spheroidal
*S. rectilineum*	5 (2500)	97.68	5 (100)	28.56	31.53	0.91	oblate spheroidal
*S. restioides*	1 (500)	97.80	1 (20)	34.60	35.70	0.97	oblate spheroidal
*S.* sp. nov. aff. *alatum*	1 (500)	22.70	1 (20)	44.90	43.30	1.04	prolate spheroidal
*S. weirii*	1 (500)	93.50	1 (20)	35.90	41.60	0.86	suboblate
*S. wettsteinii*	5 (2500)	93.72	5 (100)	29.48	32.59	0.90	oblate spheroidal

Notes:^*^N, number of individuals analyzed (number of cells). ^#^Percentage of normal cells.

### Mitotic analysis

Root tips were pretreated with 8-hydroxyquinoline (2 mM) for 24 h at 10 °C, fixed in absolute ethanol/glacial acetic acid (3:1, v/v) for 24 h at room temperature and stored at -20 °C until further analysis. Fixed root tips were washed in 10 mM citrate buffer pH 4.6 and digested in a solution of 4% cellulase, 1% pectolyase and 4% hemicellulase at 37 °C for about 30 min. Digested root tips were macerated in a drop of 45% acetic acid and slides were coverslipped; coverslips were removed in liquid nitrogen and the slides were air dried. The best slides were stained in 2% Giemsa and mounted in Entellan (Merck). Alternatively, some slides were prepared following the standard Feulgen method. All observations were performed using a Zeiss Axioplan Universal photomicroscope.

### Genome size estimations

Total DNA content was assessed by flow cytometry according to Marie and Brown (1993) and Dolezel *et al.* (2003). *Petunia hybrida* Vilm. ‘PxPC6’ (2C = 2.85 pg; Marie and Brown, 1993), *Solanum lycopersicum* L. ‘Montfavet 63-5’ (2C = 1.99 pg, Lepers-Andrzejewski *et al.*, 2011) and *Pisum sativum* L. ‘Long Express’(2C = 8.37 pg, Marie and Brown, 1993) were used as internal standards. To release the nuclei into suspension, the material was chopped in 1 mL of a nuclear-isolation buffer (Galbraith *et al.*, 1983) supplemented with 10 mM sodium metabisulphite and 1% polyvinylpyrrolidone 10000 and RNase (2.5 U/mL; Amresco, USA). The DNA content of 5,000–10,000 stained nuclei was determined for each sample using either an Elite ESP (Beckman-Coulter, Brea, CA, USA), a Partec CyFlow or a FACSAria II (Becton Dickinson, Franklin Lakes, NJ, USA) flow cytometer. For each measurement of DNA content, 3–5 samples were assessed and the average value was used as the 2C content for the following analyses. The total 2C DNA value was calculated as: sample peak mean / standard peak mean × 2C DNA content of standard (pg). The term ‘monoploid genome size’ (1C*x*) was used to represent the DNA content of one non-replicated genome with basic chromosome number *x* (Greilhuber *et al.*, 2005), whereas 2C refers to the whole GS of a somatic cell.

### Meiotic analysis

Flower buds were fixed in ethanol/glacial acetic acid (3:1, v/v) for 24 h at room temperature and kept at -20 °C. For slide preparation, anthers were washed in distilled water and squashed in 1% propionic carmine. Slides were examined and documented with an Axioplan Universal photomicroscope (Zeiss, Oberkochen, Germany). All available phases of meiosis I and II were analyzed. Abnormalities, such as non-orientated bivalents and multivalents in metaphase I, bridges and laggards in anaphase and telophases I and II, were evaluated. Meiotic indexes were calculated from 200 pollen tetrads per plant using the formula: MI = (number of normal tetrads/total number of tetrads) × 100. Microcytes and micronuclei, bridges, and unequally sized cells were considered abnormalities. Chromosome numbers were determined in diakinesis (prophase I).

### Pollen stainability and morphology

Pollen stainability and pollen morphology were used to assess pollen viability. Flowers buds at preanthesis were collected, fixed and stored as described above. Slides were prepared following Alexander’s method (Alexander, 1980), in which empty, non-viable pollen grains stain green, whereas full, viable pollen grains stain purple. Samples of 500 pollen grains per flower were analyzed from at least one individual per taxon. In order to determine pollen grain shape, measurements of the polar axis (P) and equatorial diameter (E) of 20 mature pollen grains per individual were performed after Alexander’s staining method. The P/E ratios of the grains were used to classify pollen morphology according to Erdtman (1971).

### Statistical analysis

The DNA content 2C and 1C*x* were compared between diploids and polyploids using Student’s *t*-test, whereas one-way ANOVA followed by Tukeys multiple comparisons tests were employed to compare taxa for these variables. Data of one population, at random, was used when data of more than one population was available for a determined taxon. The ploidy level of two taxa (*S. brasiliense* and *S. caeteanum*) of unknown chromosome number was inferred taking into account their 2C content, using two approaches: a cluster analysis using squared Euclidian distance and centroid linkage in order to group taxa, and a discriminant analysis, using prior membership probabilities based on the number of known diploid and polyploid species (13 and 3 species, respectively). A p-value of 0.05 was considered the threshold for statistical significance.

### Sequence data, alignments and phylogenetic analyses

Newly sequenced specimens were field-collected and genomic DNA was extracted from 10–15 mg silica-dried leaf material using a modified CTAB protocol with volumes adjusted to 2 mL tubes (Doyle and Doyle, 1990). A combination of nine coding and non-coding DNA plastid, mitochondrial and nuclear regions previously used by Chauveau *et al.* (2011, 2012) was selected (i.e. *matK*, *rpoC1*, *rpoB*, *matK-5’trnK*, *psbA-trnH*, *trnQ-rps16*, *nad1-2/3*, *nad4-1/2* and ITS). DNA primers and PCR amplification protocols were the same as those described in Chauveau *et al.* (2011, 2012). All PCR products were sent to the Molecular Biology and Genetic Engineering Centre of the State University of Campinas (CBMEG/UNICAMP, SP, Brazil) for sequencing. CodonCode Aligner 6.0.2 (CodonCode Corp., Dedham, MA, USA) was used to edit chromatograms and contigs. Nineteen new DNA sequences were generated for this study and sequences already deposited in GenBank were added to complete our data set. Alignments of DNA sequences were conducted with MAFFT 7 (Katoh and Standley, 2013) and manually validated with MEGA6 (Tamura *et al.*, 2013). Unambiguously aligned gaps shared by two or more taxa were coded with SeqState 1.4.1 (Müller, 2005), according to the Modified Complex Indel Coding approach (Simmons *et al.*, 2007).

Phylogenetic analyses were conducted using two parametric methods: Maximum Likelihood (ML) with bootstrapping in RAxML 8.2.9 (Stamatakis, 2014) and Bayesian inference (BI) with MrBayes 3.2.6 (Ronquist *et al.*, 2012). ML and BI analyses were initially performed on each DNA region separately, and then on cpDNA and mtDNA markers respectively combined, to detect potential outliers or incongruence among loci or genomes. Conflicts were explored through visual examination of resulting ML trees and comparison of nodes with ≥ 70% bootstrap support. These separate analyses were all conducted with RAxML as implemented on the XSEDE server of the CIPRES Science Gateway (Miller *et al.*, 2010) following a three-step process: (1) a thorough ML search with 200 randomized starting trees to find the best-scored likelihood tree; (2) non-parametric bootstrap statistics (-b) calculated from 1,000 thorough bootstrap iterations; and (3) bootstrap values were mapped on the best-scored tree. A GTRGAMMA model was used in each step with data partitioned for each gene, intron or spacer region, and codon position in the regions coding for proteins to accommodate locus-specific variations. Gap-coded characters were also partitioned by locus. All data were then combined for subsequent analyses. The Maximum Likelihood analysis of the total evidence data set was performed following the three-step process previously described, whereas partitioned MrBayes analysis was conducted with two independent runs of four chains each and eight million generations, sampling trees every 1,000 generations, using the models selected with the Akaike information criterion (AIC) by MrModeltest 2.3 (Nylander, 2004) for each partition (Table S2). Gap-coded characters were included as additional datatype and treated using a simple model with variable rates. Convergence was verified by checking the average deviation of split frequencies (< 0.01), the Effective Sample Size (ESS > 200) and the Potential Scale Reduction Factor (0.99 < PSRF < 1.01) reported by MrBayes. Default value was used for the burn-in phase and a majority-rule consensus tree was computed. Phylogenetic trees resulting from ML and BI analyses (ML best-scoring tree and BI majority-rule tree) were rooted on *S. macrocarpum* + *S. striatum* and combined to manually build a highly conservative consensus tree that summarizes the results of both analyses at once. A given node was kept in the consensus tree only if the ML bootstrap support was ≥ 70% or if the PP was ≥ 0.95 and in the absence of topological conflict among ML and BI trees.

### Ancestral character state reconstructions and regression analyses

**Figure 1:**
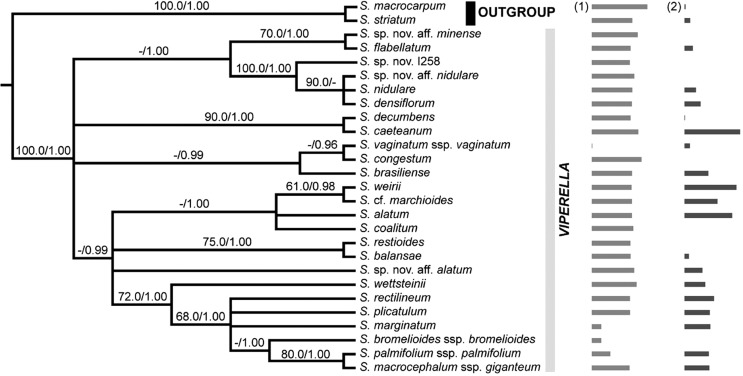
Strict consensus tree based on the estimated maximum likelihood tree and the Bayesian 50% majority rule consensus tree obtained from the analyses of the whole molecular data set. The tree is rooted using *Sisyrinchium striatum+S. macrocarpum* as outgroup. Support values indicated above branches follow the order likelihood bootstrap support (LBS)/ Bayesian posterior probability (PP) and are provided for a given node only if one of the values reached the following thresholds: LBS ≥ 70% or PP ≥ 0.95. A dash (-) indicates support value of less than 50% for LBS or less than 0.95 for PP. For each taxon, (1) horizontal light grey bars indicate normalized natural log values of altitude, whereas (2) horizontal dark grey bars show normalized DNA content (2C) values.

The consensus topology (Figure 1) was used for discrete character optimization (i.e., chromosome number) with the maximum parsimony (MP) and ML methods implemented in MESQUITE 3.10 (Maddison and Maddison, 2016). With MP, character states were treated as unordered, allowing any transition among states. ML optimization was conducted using the MK1 model of evolution (Schluter *et al.*, 1997; Pagel, 1999), which gives equal probability for changes between all character states. For continuous character optimizations (i.e., DNA content (2C) and altitude) a pruned phylogram was generated by reducing the phylogeny obtained in the current study to terminals for which we had genome size data. Maximum likelihood ancestral state reconstructions were performed using the contMap command of phytools package (Revell, 2012) in R 3.4.1 (R Core Team, 2017).

We used a multiple regression-type approach to test for adaptive adjustment of the genome size to elevation. The altitude data were log transformed and the pruned phylogram was used for phylogenetic generalized least squares (PGLS) regressions. A non-phylogenetic linear least squares regression analysis was performed in R and, to account for dependence of species values due to phylogenetic relatedness, PGLS regressions were conducted with the packages nlme (Pinheiro *et al.*, 2017) and caper (Orme *et al.*, 2013) in R. Analyses were performed with different alternative evolutionary models: Pagel’s λ (Pagel, 1997) with constraint and unconstraint values, Ornstein-Uhlenbeck (Hansen, 1997) and a Brownian motion model (Felsenstein, 1985).

## Results

### Chromosome numbers, karyotypes and genome size

Chromosome counts for 17 taxa are presented here in Table 2 (excluding outgroup species); all but one (*S. alatum*) had their number determined for the first time. Most taxa are diploids (2*n* = 2*x* = 18), and only three of them are polyploids (*Sisyrinchium alatum* and *S*. cf. *marchioides* are 2*n* = 4*x* = 36 and *S. weirii* is 2*n* = 6*x* = 54). Despite this ploidy variation, all taxa had the same base chromosome number *x* = 9. Intraspecific polyploidy or cytotype variation were not found for any species.

**Figure 2:**
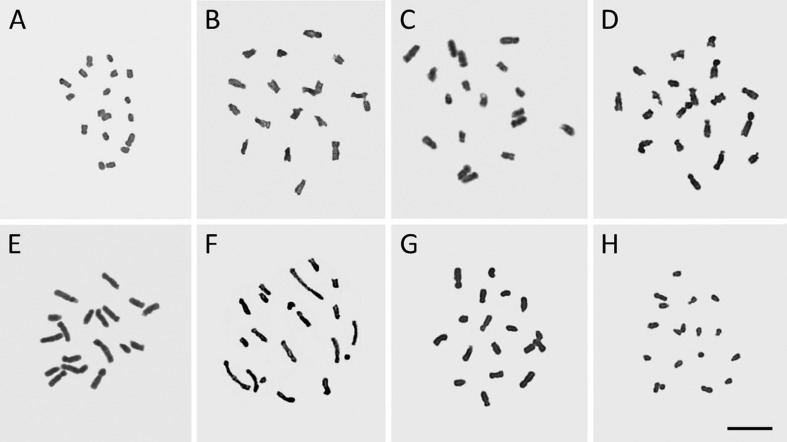
Mitotic metaphases/prometaphase of *Sisyrinchium* taxa. A, *S. decumbens*; B, *S. densiflorum*; C, *S. flabellatum*; D, *S. marginatum*; E, *S. macrocephalum* subsp. *giganteum*; F, *S. palmifolium* subsp. *palmifolium*; G, *S. rectilineum*; H, *S. wettsteinii*. Note that all have the same 2*n* = 18, but present remarkably different karyotypes in regards to chromosome size and shape (p.e. A × E). Satellites are only recognizable in E and F. Bar in H represents 10 μm.

Although chromosome measurements were not carried out in any species, a remarkable difference in the size of chromosomes could be observed among the analyzed taxa. The smallest chromosomes were those of *S. decumbens* and *S. wettsteinii* (Figure 2A and H, respectively), and the largest chromosomes were found in *S. macrocephalum* subsp. *giganteum* (Figure 2E). With regard to chromosome size, *Sisyrinchium decumbens* and *S. densiflorum* (Figure 2A and B, respectively) apparently have more symmetrical karyotypes, while, *S. macrocephalum* subsp. *giganteum* and *S. palmifolium* subsp. *palmifolium* seemed to have the most asymmetrical karyotypes, comprising large and small chromosome pairs. The first one has at least three pairs of satellited chromosomes and the second species with one satellite pair was clearly observed (Figure 2E and F respectively).

**Figure 3:**
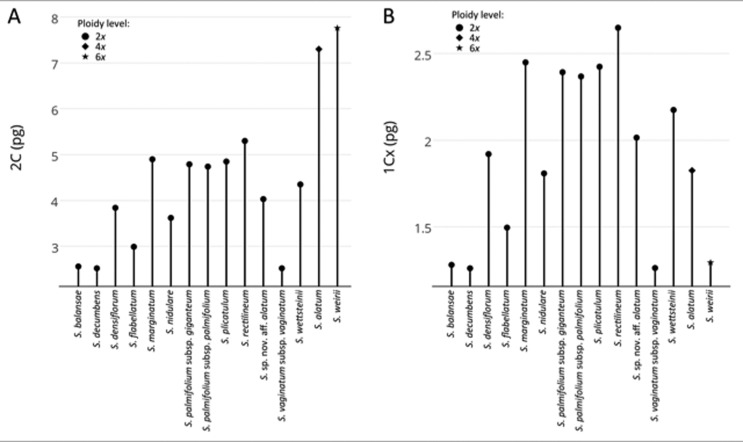
Distribution of DNA content (2C) (A) and monoploid genome size (1C*x*) (B) among *Sisyrinchium* taxa. Ploidy levels are indicated as circle (2*x*), star (4*x*) or hexagon (6*x*).

Concerning DNA amount, we observed 2C ranging from 2C = 2.08 pg in *S. decumbens* to 2C = 8.17 pg in *S. caeteanum* (Table 2, Figure 3A), representing a difference of more than 3-fold in genome size. As expected, the 2C values increased according to ploidy level (*t* = 4.67, *p* < 0.001, n = 13 diploid and 3 polyploid species), with smaller DNA content in diploids (mean ± SD: 3.92 ± 0.99; n = 13 species) and higher in the polyploids (tetraploids *S.* cf*. marchioides* 2C = 5.69 and *S. alatum* 2C = 7.30 pg*;* hexaploid *S. weirii* 2C = 7.77 pg; mean ± SD: 6.92 ± 1.09; n = 3 species). Although *S. caeteanum* presented the largest genome (8.17 pg), compared to the other species, its chromosome number and ploidy could not be ascertained. On the other hand, taking into account only the 13 diploid taxa (all 2*n* = 18), the DNA content varied greatly, ranging from 2C = 2.08 to 5.30 pg, evidencing an increase in 2C DNA content not associated with the ploidy level (Figure 3A). Monoploid genome sizes (1C*x*) ranged from 1.04 pg to 2.65 pg (Table 2, Figure 3B). Tetraploids had 1C*x* values in average lower than most diploid taxa (mean ± SD: 1.62 ± 0.29; n = 2 species), and the hexaploid showed one of the smallest 1C*x* (1.16 pg) (see Figure 3B). However, the average difference in C*x* between diploids and polyploids was not statistically significant (*t* = 1.49, *p* = 0.159).

Chromosome number could not be determined for *Sisyrinchium brasiliense* and *S. caeteanum*, therefore the 1C*x* value could not be calculated either. Aiming to infer the ploidy level of these taxa, we performed a discriminant analysis and constructed a dendrogram based on the 2C content of all taxa. The discriminant function was statistically significant (*p* < 0.001), and all but one taxa of known ploidy were correctly classified. The exception was the tetraploid *S.* cf*. marchioides,* which was classified as diploid due to its intermediate 2C value (5.69 pg). This taxon grouped with diploids in the cluster analysis also (Figure S1). Based on the discriminant analysis, the diploid group membership probabilities for the two species of undetermined ploidy were 0.975 for *S. brasiliense* and 0.001 for *S. caeteanum*. Therefore, even though we were unable to determine chromosome numbers of *S. brasiliense* and *S. caeteanum*, the statistical analysis of genome sizes indicates that they probably are diploid and polyploid (possibly tetra- or hexaploid), respectively.

### Meiotic behavior and pollen viability/morphology

**Figure 4:**
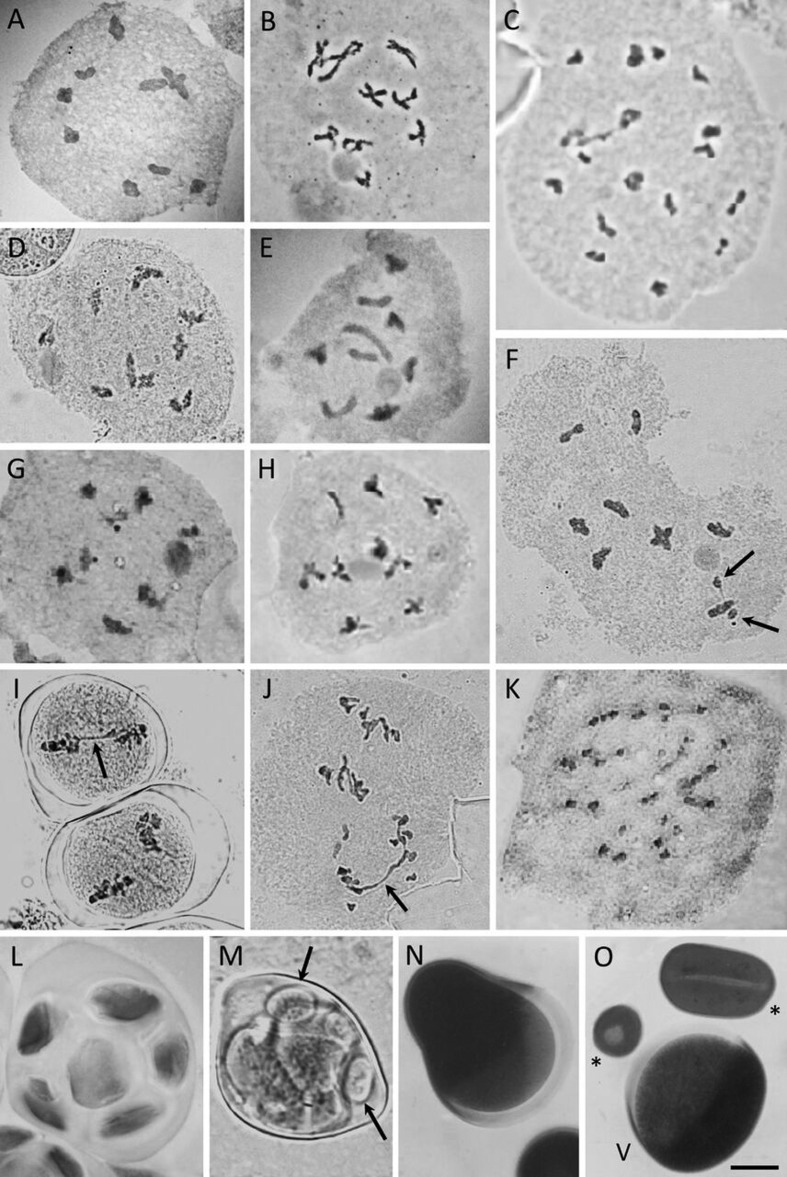
Meiotic analysis of *Sisyrinchium* taxa – haploid chromosome numbers and observed meiotic abnormalities. A, *S. palmifolium* subsp. *palmifolium*, *n* = 9 (prophase I - diakinesis); B, *S. marginatum*, *n* = 9 (prophase I - diplotene); C, *S. alatum*, *n* = 18 (prophase I - diakinesis); D, *S. decumbens*, *n* = 9 (prophase I - diakinesis); E, *S. macrocephalum* subsp*. giganteum*, *n* = 9 (prophase I - diakinesis); F, *S. rectilineum*, *n* = 9, in diakinesis with two univalents (arrows); G, *S*. sp. nov. aff. *alatum*, *n* = 9 (prophase I - diakinesis); H, *S. restioides*, *n* = 9 (prophase I - diakinesis); I, *S. rectilineum* in metaphase I with stickiness (arrow); J, *S. rectilineum* in anaphase II with bridge (arrow); K, *S. weirii*, *n* = 27 (prophase I - diakinesis); L, *S. palmifolium* subsp. *palmifolium* showing ‘tetrad’ with several microspores; M, *S. rectilineum* showing tetrad with microcytes (arrows); N, *S. marginatum* showing an abnormally shaped pollen grain; O, *S. marginatum* showing viable pollen grain (V) and two unviable pollen grains (*). Bar in O represents 10 μm.

All taxa presented highly regular meiosis (97.11–99.45%; Table S1; Figure 4). The most common abnormalities were non-oriented bivalents, metaphases with stickiness (Figure 4I) and anaphase bridges (Figure 4J) in either anaphase I or anaphase II, sometimes with fragments or laggards. Multivalents and univalents were not observed in any taxa. The meiotic indexes were high for all taxa (> 95%; Table S1) and they had at least 87% viable pollen grains (Table 3), except for *Sisyrinchium* sp. nov. aff. *alatum* which had a very low pollen viability (22.70%). Pollen grains were classified into three distinct morphologies according to their P/E ratio (Table 3). We found that most taxa have oblate spheroidal pollens, while only one species (*Sisyrinchium* sp. nov. aff. *alatum*) has prolate spheroidal pollen grains. The smallest pollen measures were observed in *S. balansae* and the largest were those from *S.* sp. nov. aff. *alatum,* both diploid species.

### Phylogenetic analyses

Since no significantly supported incongruence was detected among tree topologies obtained from independent analyses of each DNA marker (data not shown), all DNA regions from the three genomic compartments were concatenated for subsequent analyses. The combined alignment reached 8565 characters divided into 8535 nucleotide positions and 30 coded indels. Plastid, mitochondrial and nuclear regions contained, respectively, 4639, 3272 and 684 characters. The number of potentially informative sites was 123 (2.65%) for the cpDNA data set, 70 (2.14%) for the mtDNA matrix and 54 (7.89%) for the ITS alignment. ML and Bayesian analyses of the total combined data set produced similar topologies. Therefore, the ML best-scored tree and the 50% majority-rule BI tree were summarized as a strict consensus phylogeny presented in Figure 1, with nodes supported above 70% by ML bootstrap estimates or 0.95 by BI posterior probability values.

### Ancestral character state reconstructions and regression analyses

**Figure 5:**
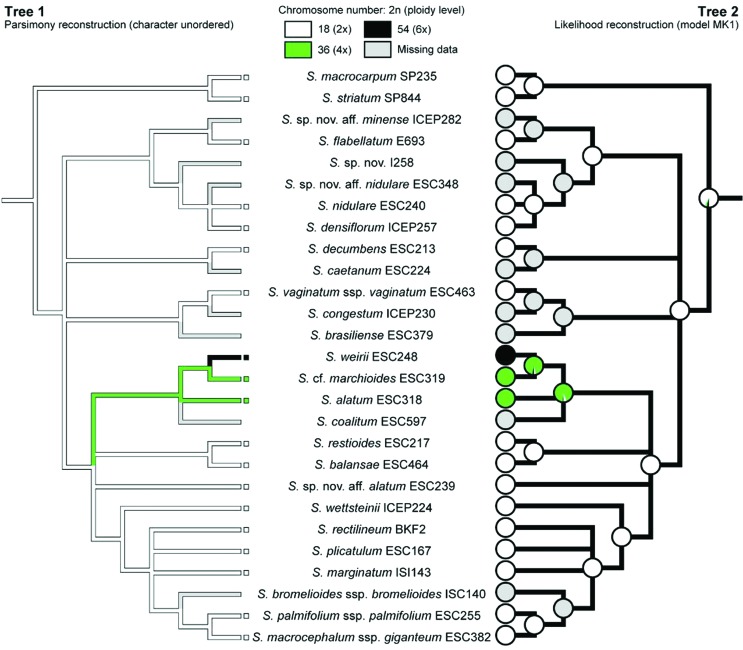
Mirror trees showing MP (Tree 1) and ML (Tree 2) optimizations of diploid chromosome numbers (2*n*) on the strict consensus tree. External nodes are coloured according to the character state observed, whereas internal nodes are coloured according to the ancestral state inferred (Tree 1) or to the relative likelihood values calculated for each character state (Tree 2). Missing data are indicated in grey.

The MP and ML analyses of the diploid chromosome number (2*n*) evolution on the strict consensus tree produced consistent results (Figure 5), and both phylogenetic uncertainty and missing data had little effect on reconstructed ancestral states at key nodes. Character optimizations suggested that the common ancestor of sect. *Viperella* was probably diploid (2*n* = 2*x* =18), with two subsequent polyplodization events within the section. One transition towards tetraploidy occurred at the base of the clade formed by *S. coalitum*, *S. alatum*, *S.* cf. *marchioides* and *S. weirii,* and the hexaploid condition of the latter species was probably derived from the common tetraploid ancestor of *S.* cf. *marchioides* + *S. weirii*.

**Figure 6:**
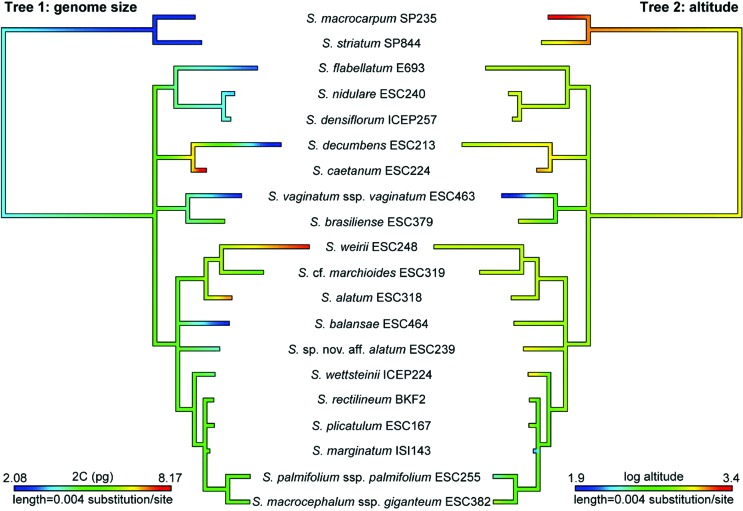
Mirror trees showing maximum likelihood ancestral state reconstructions of DNA content (2C) on Tree 1 and natural log of altitude on Tree 2. Optimizations of continuous characters were performed with the contMap command of Phytools in R. Branch lengths are proportional to the number of molecular substitutions per site.

Total DNA content (2C) varied considerably and repeatedly across the phylogeny (Figure 6, Tree 1), with conspicuously larger genomes in polyploid species and in *S. caeteanum*. ML optimization of genome size evolution suggested that the section’s ancestral state was intermediate (2C of ca. 4.72 pg) and was relatively higher at the base of *S. alatum* + *S.* cf. *marchioides* + *S. weirii* (2C of ca. 6.05 pg) and the clade formed by *S. caeteanum* and *S. decumbens* (2C of ca. 6.65 pg). Altitude also varied repeatedly across the phylogeny (Figure 6, Tree 2), and the ML optimization of this environmental factor along the phylogeny showed that the ancestor of *Viperella* probably occurred at ca. 790 m elevation. However, the ancestral state analysis did not reveal a specific pattern of evolution in relation to genome size.

Significant phylogenetic signals were not detected for both genome size (2C) and altitude when measured using Pagel’s λ and Blomberg’s *K* (Table 4). Moreover, results of non-phylogenetic linear least squares regression and phylogenetic generalized least squares (PGLS) regressions (Table 5) showed that no significant relationship (*p* > 0.05) was found between the magnitude or direction of DNA content changes and natural log of altitude in our data set.

**Table 4:** Tests for phylogenetic signal of DNA content (2C) and altitude (natural log; m) among taxa of *Sisyrinchium* used in the present study.

	*K*	*P-value*	λ	*P-value*
Genome size	0.487	0.449	0.457	0.415
log of altitude	0.134	0.533	6.6*10^-5^	0.691

*Notes:* Blomberg’s K and Pagel’s λ were calculated using the phylosig command in phytools (Revell, 2012) in R.

**Table 5:** Tests for adaptive adjustments of the DNA content (2C)-altitude (natural log; m) relationship among taxa of *Sisyrinchium* used in the present study: results from the non-phylogenetic linear least squares regression and phylogenetic generalized least squares (PGLS) regressions.

	AIC	Slope	Std. error	*P-value*
Linear regression	NA	0.504	1.247	0.690
PGLS				
Brownian motion	81.632	0.504	0.651	0.449
Ornstein-Uhlenbeck	81.385	0.544	0.741	0.472
Pagel’s λ:				
λ=0	82.428	0.504	1.247	0.690
λ=1	77.465	0.504	0.651	0.449
λ unconstraint	81.418	0.964	1.111	0.397

## Discussion

### Karyotype and GS evolution

The data obtained here demonstrate that base chromosome number *x* = 9 is constant among taxa of sect. *Viperella* which is also the most frequent in Southern Hemisphere *Sisyrinchium* species (Kenton and Heywood, 1984). Thus, this is a constant character within the clade, in which the absence of dysploidy and few polyploidy events were observed. Diploid chromosome number (2*n* = 18) was estimated as the ancestral condition of the section and polyploidy is a derived character restricted to only three species (Figure 5), suggesting that this phenomenon was not important to the evolution of this group, contrary to almost all other Iridoideae from South America (Moraes *et al.*, 2015). Notwithstanding the role of polyploidy in Iridaceae evolution, diploids and lower polyploids (tetra- and hexaploids) are most frequent among Southern Hemisphere *Sisyrinchium* (Tacuatiá *et al.*, 2012, 2016). The optimization of ploidy level shown in Figure 5 evidenced that a polyploidization event gave rise to the tetraploid *S. alatum* and *S.* cf*. marchioides* and the hexaploid *S. weirii*; while, the hexaploid condition was probably derived from the common tetraploid ancestor of *S.* cf. *marchioides* + *S. weirii,* as was already proposed here.

Polyploid cytotypes have been reported in some Iridaceae species, such as *S. micranthum* (4*x* and 6*x*; Tacuatiá *et al.*, 2012), *S. sellowianum* Klatt (4*x*; Fachinetto *et al.*, 2018) and *Herbertia lahue* (Molina) Goldblatt (4*x*, 6*x* and 8*x*; Moraes *et al.*, 2015; Stiehl-Alves *et al.*, 2016). However, in this study we did not find any cytotypes within taxa. Different populations/localities (see Tables 1 and 2) were sampled for *S. decumbens, S. marginatum, S. vaginatum* subsp. *vaginatum* and *S. palmifolium* subsp. *palmifolium*, but different chromosome numbers were not found in any accessions for these taxa. Hence, events of intraspecific polyploidization apparently did not take place within taxa of sect. *Viperella*.

A noticeable chromosome size variation was observed among the studied *Viperella* taxa, as previously reported for other South American species of *Sisyrinchium* (Kenton and Heywood, 1984; Rudall *et al.*, 1986). Even without chromosome measurements performed for such species, it is possible to see that *Sisyrinchium decumbens* presents the most symmetrical karyotype and has also the smallest DNA content, while *S. palmifolium* subsp. *palmifolium* has a more asymmetrical karyotype and a considerably higher DNA amount. This pattern has been reported across Liliaceae (Peruzzi *et al.*, 2009), indicating that increases in genome size were generally accompanied by increasing karyotype asymmetry. In future studies, it might be interesting to test the association between the symmetry and the homogeneity of certain environmental variables, such as climatic or geographic parameters.

In relation to the character genome size, an intermediate 2C value was the ancestral state from which genome size increases and decreases took place, as is remarkable for *S. decumbens.* Probably, all these transitions could be interpreted as independent or homoplasious events and thus, the increment of genome size does not necessarily correspond to speciation events in this clade. Considering the occurrence of only three polyploid species in this section, it seems that the transitions towards larger genome size are not related to polyploidy.

Although Iridaceae presents a large range of 2C values from 0.96 to 62.76 pg (Bennett and Leitch, 2012), *Sisyrinchium* is known to display much smaller values, that vary between 2C = 1.00 and 2C = 8.40 pg and a monoploid genome size (1C*x*) ranging from 0.32 to 3.56 pg (Kenton *et al.*, 1986). Estimates of DNA amounts for *Sisyrinchium* species are reported solely for 30 taxa, less than 22% of the total number of species in the genus (Kenton *et al.*, 1986). All taxa examined in the present work, except *S. alatum,* had their genome size estimated for the first time. According to the size categories established by Leitch *et al.* (2005), most of the investigated *Sisyrinchium* representatives (12 species; 60%) fall within the category of small genome sizes (2.8 ≤ 2C < 7pg); five species (25%) have very small (2C < 2.8) and only three present intermediate (7 ≤ 2C < 28) genome sizes. In our study, taxa of sect. *Viperella*, although with variable genome sizes, present the same base chromosome number (*x* = 9), as seen in sect. *Echthronema* (*sensu* Benth. & Hook.) (Kenton *et al.*, 1986). Despite such relationships and similarities, the analyzed taxa differed greatly in their genome sizes, especially considering the three polyploid species.

Taxa studied herein presented intermediate to high 1C*x* values, with the smallest genomes in *S. decumbens* (1C*x* = 1.08 pg) and the largest in *S. rectilineum* (1C*x* = 2.65 pg), both diploids. Considering that all diploid taxa have the same chromosome number (2*n* = 18), it is noteworthy that the genome size of *S. decumbens* is approximately half of that found in *S. rectilineum*. Compared to diploids, the polyploids *S. alatum*, *S.* cf*. marchioides* and *S. weirii* presented a reduction in 1C*x*, which is even higher in the hexaploid *S. weirii*. Such reduction could be a ‘genome downsizing’ effect, as widely reported for many polyploid species (Leitch and Bennett, 2004; Suda *et al.*, 2007; Pellicer *et al.*, 2010), including *Sisyrinchium* (Kenton *et al.*, 1986; Tacuatiá *et al.*, 2016). Although the difference in 1C*x* content between diploids and polyploids was not statistically significant, our data suggest that genome downsizing may also be present in the section *Viperella,* but was not detected statistically, probably due to the small number of polyploid taxa. It has been reported that ploidy level, chromosome size and genome size increase with latitude and/or altitude for several *Sisyrinchium* species (Rudall *et al.*, 1986; Kenton *et al.*, 1986). In our study, variability in genome size could not be attributed to altitude, once significant phylogenetic signals were not detected in the regression analysis (see Table 5 and Figure 6), in spite of the occurrence of all polyploid species in altitudes higher than 900 m (Table 1).

Different proportions of repetitive DNA sequences, especially transposable elements (TE), can also cause genome size variation in plants (Bennetzen *et al.*, 2005; Leitch and Leitch, 2012). Thus, differential TE activity might be a mechanism behind 2C content variation found in diploids from our data set. Slow growing, long lived species might contain greater accumulation of repetitive sequences than faster developing species (Charlesworth *et al.*, 1994), providing an explanation for the larger genomes of the perennial taxa studied here compared to the annual species investigated by Tacuatiá *et al.* (2016). Thus, future work aiming repetitive DNA sequences characterization in this group of species should be done.

### Meiotic behavior and pollen viability

The meiotic behavior and pollen viability data obtained here are relevant to the investigation of the mechanisms of polyploidy involved in the evolutionary process. As expected, almost all diploid taxa presented a regular meiosis and high pollen viability. Although meiotic behavior and meiotic indexes were not analyzed for polyploid species, elevated pollen viability was found in *S. alatum* (98.50%, 2n = 4x = 36) and *S. weirii* (93.5%, 2n = 6x =54), indicating meiotic regularity and male-fertile plants. Such stable behavior was also reported for *S. micranthum*, in both diploids and polyploids (Tacuatiá *et al.*, 2012) and for other *Sisyrinchium* species that were considered interspecific hybrids that underwent genome stabilization (Henderson, 1976; Cholewa and Henderson, 1984). The diploid *Sisyrinchium* sp. nov. aff. *alatum* was the only species with very low pollen viability (22.70%), and in this case a putative hybrid status cannot be ruled out. More morphological and cytological studies are necessary to confirm this assumption.

Significant differences on pollen size were found among cytotypes of *S. micranthum* (Tacuatiá *et al.*, 2012), where hexaploids (2*n* = 48) presented pollen grains significantly bigger than those of diploids (2*n* = 16). In the present work, although there was no apparent correlation between 2C DNA contents and pollen grain size, polyploid species have some of the largest pollen grains and also the highest 2C DNA contents (Tables 2 and 3).

### Concluding remarks

This study reports the first attempts to understand patterns underlying karyotype and genome evolution in *Sisyrinchium,* sect. *Viperella.* Despite the stability in base chromosome number (*x* = 9), evolutionary changes in karyotypes of this section involve primarily variation in DNA amount, regardless of ploidy level, and secondly, polyploidy and variation in chromosome size. Although polyploidy and disploidy are considered important factors in the evolution of South American Iridaceae, in *Sisyrinchium* sect. *Viperella* polyploids they are less frequent, and disploidy changes were not reported. By raising mostly novel cytological data and analyzing them in a phylogenetic context, we suggest six transitions toward a genome size increase for the taxa of sect. *Viperella* that were originated from mechanisms other than polyploidy.

## Supplementary material

The following online material is available for this article:

Table S1 - Data of meiotic behaviour and meiotic index.

Table S2 - Dataset partitions for Maximum Likelihood (ML) and Bayesian Inference (BI) analyses.

Figure S1 - Cluster analysis by Centroid Linkage method grouping taxa according to 2C genome size.
