# Supplementary material for: Cytological and genome size data analyzed in a phylogenetic frame: Evolutionary implications concerning Sisyrinchium taxa (Iridaceae: Iridoideae)
Source: Genet Mol Biol. 2018 Mar 1;41(1 Suppl 1):288–307. doi: 10.1590/1678-4685-GMB-2017-0077 (PMC5913718; doi:10.1590/1678-4685-GMB-2017-0077)
Supplement: Supplementary file 1 [file 1415-4757-GMB-41-01-2017-0077-s001.pdf]

# Supplementary Material to “Cytological and genome size data analyzed in a phylogenetic frame: evolutionary implications concerning Sisyrinchium taxa (Iridaceae: Iridoideae)”

**Table S1:** Haploid chromosome number, meiotic normality and meiotic indexes in analyzed species of *Sisyrinchium.*

| Species | *n* | Meiosis I and II | | Meiotic index | |
|---|---|---|---|---|---|
| | | N* | %‡ | N* | %‡ |
| *S. decumbens* | 9 | 17 (4474) | 97.81 | 5 (906) | 98.90 |
| *S. macrocephalum* ssp. *giganteum* | 9 | 5 (550) | 99.45 | 4 (800) | 99.63 |
| *S. marginatum* | 9 | 6 (3357) | 98.18 | 9 (2456) | 99.47 |
| *S. palmifolium* ssp. *palmifolium* | 9 | 9 (2074) | 97.11 | 9 (2800) | 95.21 |
| *S. plicatulum* | 9 | 1 (291) | 97.94 | — | — |
| *S. rectilineum* | 9 | 3 (1552) | 97.94 | 3 (600) | 99.50 |
| *S. wettsteinii* | 9 | 6 (692) | 98.12 | 9 (1801) | 99.61 |

*Notes:* *N, number of individuals analyzed (number of cells).‡Percentage of normal cells.
