# Supplementary material for: Cytological and genome size data analyzed in a phylogenetic frame: Evolutionary implications concerning Sisyrinchium taxa (Iridaceae: Iridoideae)
Source: Genet Mol Biol. 2018 Mar 1;41(1 Suppl 1):288–307. doi: 10.1590/1678-4685-GMB-2017-0077 (PMC5913718; doi:10.1590/1678-4685-GMB-2017-0077)
Supplement: Supplementary file 2 [file 1415-4757-GMB-41-01-2017-0077-s002.pdf]

Supplementary Material to "Cytological and genome size data analyzed in a phylogenetic frame: evolutionary implications concerning Sisyrinchium taxa (Iridaceae: Iridoideae)"

Table S2: Dataset partitions for Maximum Likelihood (ML) and Bayesian Inference (BI) analyses and evolutionary models used in BI

Data partition	No. of positions (without primers)	Partition by codon position	Model
cpDNA partition			
rpoC1 (partial sequence)	508	X	HKY
rpoB (partial sequence)	472	X	HKY+I
matK (partial sequence)	1572	X	GTR+I
matK-5'*trnK* intron (complete sequence)	242		F81
psbA (partial sequence)	53	X	K80
psbA-*rps19* spacer (complete sequence)	124		HKY
rps19 (complete sequence - negative strand)	279	X	HKY
rps19-*trnH* spacer (complete sequence)	137		F81
trnQ (partial sequence)	49		K80
trnQ-*rps16* spacer (partial sequence)	1185		GTR
mtDNA partition			
5'-nad1 exon 2 (partial sequence)	57		HKY
nad1 exon 2 (complete sequence)	82	X	HKY
nad1 intron 2 (complete sequence)	1458		HKY
nad4 intron 1 (complete sequence)	1386		GTR+I
nad4 exon 2 (partial sequence)	271	X	HKY
nuDNA partition			
Internal Transcribed Spacer 1 (complete sequence)	232		GTR+Γ
5.8S ribosomal RNA (complete sequence)	164		K80
Internal Transcribed Spacer 2 (complete sequence)	246		HKY+I
28S ribosomal RNA (partial sequence)	42		JC

Notes: partition by codon position = partition coding for protein.
