# Supplementary material for: Cytological and genome size data analyzed in a phylogenetic frame: Evolutionary implications concerning Sisyrinchium taxa (Iridaceae: Iridoideae)
Source: Genet Mol Biol. 2018 Mar 1;41(1 Suppl 1):288–307. doi: 10.1590/1678-4685-GMB-2017-0077 (PMC5913718; doi:10.1590/1678-4685-GMB-2017-0077)
Supplement: Supplementary file 3 [file 1415-4757-GMB-41-01-2017-0077-s003.pdf]

Supplementary Material to "Cytological and genome size data analyzed in a phylogenetic frame: evolutionary implications concerning Sisyrinchium taxa (Iridaceae: Iridoideae)"

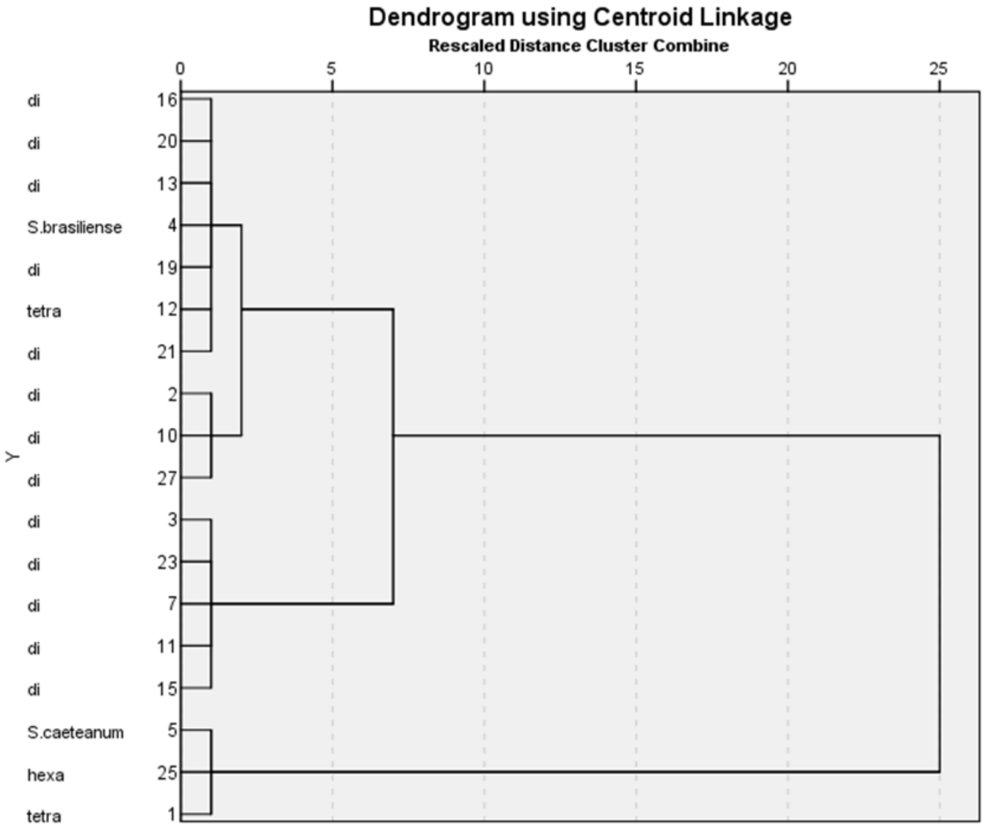

Figure S1: Cluster analysis by Centroid Linkage method grouping taxa according to 2C genome size
